# A Simple Phase-Sensitive Surface Plasmon Resonance Sensor Based on Simultaneous Polarization Measurement Strategy

**DOI:** 10.3390/s21227615

**Published:** 2021-11-16

**Authors:** Meng-Chi Li, Kai-Ren Chen, Chien-Cheng Kuo, Yu-Xen Lin, Li-Chen Su

**Affiliations:** 1 Thin Film Technology Center, National Central University, Taoyuan 320317, Taiwan; mcli@dop.ncu.edu.tw (M.-C.L.); cckuo@ncu.edu.tw (C.-C.K.); 2 Optical Sciences Center, National Central University, Taoyuan 320317, Taiwan; 3 Department of Optics and Photonics, National Central University, Taoyuan 320317, Taiwan; 110286003@cc.ncu.edu.tw; 4 TeraOptics Corporation, Taoyuan 32472, Taiwan; adderlin@teraopt.com; 5 General Education Center, Ming Chi University of Technology, New Taipei 243303, Taiwan; 6 Organic Electronics Research Center, Ming Chi University of Technology, New Taipei 243303, Taiwan

**Keywords:** phase-sensitive SPR, pixelated micropolarizer array, phase-shift interferometry

## Abstract

The SPR phenomenon results in an abrupt change in the optical phase such that one can measure the phase shift of the reflected light as a sensing parameter. Moreover, many studies have demonstrated that the phase changes more acutely than the intensity, leading to a higher sensitivity to the refractive index change. However, currently, the optical phase cannot be measured directly because of its high frequency; therefore, investigators usually have to use complicated techniques for the extraction of phase information. In this study, we propose a simple and effective strategy for measuring the SPR phase shift based on phase-shift interferometry. In this system, the polarization-dependent interference signals are recorded simultaneously by a pixelated polarization camera in a single snapshot. Subsequently, the phase information can be effortlessly acquired by a phase extraction algorithm. Experimentally, the proposed phase-sensitive SPR sensor was successfully applied for the detection of small molecules of glyphosate, which is the most frequently used herbicide worldwide. Additionally, the sensor exhibited a detection limit of 15 ng/mL (0.015 ppm). Regarding its simplicity and effectiveness, we believe that our phase-sensitive SPR system presents a prospective method for acquiring phase signals.

## 1. Introduction

Surface plasmon resonance (SPR) has become known for the rapid, sensitive, and label-free sensing of physical and (bio)chemical processes at interfaces [1,2]. The optoelectronic phenomenon refers to the observation that the free electrons on a metal surface occur with collective oscillation, resulting in the maximum absorption of incident light with a specific wavelength or at a certain incident angle [3,4]. The resonance conditions (wavelength and angle) of SPR are easily affected by the changes in the refractive index surrounding the metal surface. Such changes can be measured by the shift in the resonance angle [5,6], the resonance wavelength [7,8,9], or the intensity of the reflected light [10,11,12], which are the three commonly used types of SPR interrogation technologies. Thus, SPR sensors are frequently implemented to detect the refractive index changes caused by binding events between analytes and receptors on the metallic sensor surface, which relates to the mass deposited on the SPR sensing surface. In other words, the SPR signals are proportional to the mass change caused by the analytes. If the molecular weight of the analyte is large (i.e., higher than 1000 Da), direct detection by the SPR sensor is possible. However, the direct detection of small molecules is a challenge for the detection sensitivity of SPR sensors [13].

In addition to causing resonance angle and resonance wavelength shifts, as well as the intensity change in the reflected light, the SPR phenomenon also causes an abrupt change in the optical phase such that one can measure the phase shift of the reflected light as a sensing parameter, i.e., the SPR sensor is phase-sensitive. Phase-sensitive SPR could be a strategy for the direct detection of small molecules, given that many studies have demonstrated that the phase changes more acutely than the intensity, leading to a higher sensitivity to the refractive index change [14,15,16,17,18,19,20]. However, currently, the optical phase cannot be measured easily and directly because of its high frequency; therefore, investigators have to use some specific techniques for the extraction of phase information. Thus, phase-sensitive SPR systems usually need a phase modulator to help them extract the phase information.

In the past, electronic modulators such as piezoelectric transducers (PZT), acousto-optic modulators (AOM), electro-optic modulators (EOM), photoelastic modulators (PEM), and liquid crystal modulators (LCM) were the most widely implemented modulators in phase-sensitive SPR systems. For instance, in the Mach–Zehnder interferometer, the reference beam is introduced as a temporally modulated phase by a PZT and interferes with the signal beam that is coupled with surface plasmons, and then the SPR phase can be obtained by analyzing the interference signals [21,22]. Instead of a mechanical moving component, some groups adopted AOMs and EOMs to generate two light beams with slightly different frequencies to produce optical heterodyne signals, and the interference signal with a beat frequency can retrieve the phase difference caused by the SPR phenomenon through the use of a phase meter [23,24,25]. To simplify the structure to a single-beam configuration, many groups incorporated a PEM into the SPR system in order to introduce a sinusoidal phase modulation between the p- and s-polarizations, and determine the phase information under SPR by decomposing the detected harmonic signals [17,18,26]. An LCM is also a phase retarder that is able to induce an accurate phase shift between the p- and s-polarized components by applying appropriate voltages, and finally obtaining the SPR-induced phase difference with the use of a phase retrieval algorithm [27,28]. Recently, a few teams have also proposed SPR ellipsometry based on PEM and LCM phase modulators [29,30]. However, the use of electronic modulators could result in a significant increase in both the complexity and the cost of the system, not to mention that it may also increase noise [20]. 

In addition to the electronic modulators, there has been increasing interest in recent years in the SPR system that combines spectral interrogation with phase detection [19,20,31,32]. The optical setup is usually based on white-light interferometry, and incorporates a birefringent crystal into the SPR spectral interferometer to introduce spectral phase modulation between the two co-propagating polarization components. The phase change caused by the SPR effect is retrieved from the recorded spectral interferogram by a windowed Fourier transform. Although the systems with spectral phase modulators are set up more simply than the electronic modulator-based phase-sensitive SPR system, a delicately custom-made birefringence crystal and a complex phase-retrieve algorithm are needed [33]. On the other hand, there are also a few phase-sensitive SPR systems with no need of phase modulators. For example, one such system makes use of ellipsometry to analyze the polarization ellipse of light that experiences SPR effect by rotating analyzer [34,35,36]. Though the rotating analyzer method is performed without electronic phase modulators, it is incapable of real-time detection due to the limit of the mechanical speed.

The phase-sensitive SPR sensor has become a promising technique for optical biosensing due to its high sensitivity to refractive index changes. However, this method is not yet applied to commercial SPR instruments. This might be because of the complexity of the optical system and the data acquisition and analysis. In this study, we propose a phase-sensitive SPR sensor with a simple optical setup based on phase-shift interferometry. In this system, a pixelated polarization camera (PPC), which integrates the filters of four polarization orientations at the pixel level, is introduced to acquire the four polarization angles at the same time. Moreover, the phase information is extracted from the polarization-dependent signals recorded simultaneously by the PPC on the basis of phase-shift algorithms in a single snapshot measurement without extra electronic or mechanical modulators. Furthermore, this technique enables internal referencing to be implemented simpler than the traditional phase-sensitive SPR sensors; the internal referencing is helpful in degrading the effect of microfluctuations of temperature, which plays a key role in the SPR detection performance. Experimentally, the proposed phase-sensitive SPR sensor was applied for the detection of small molecules of glyphosate, which is the most frequently used herbicide worldwide. Traditional methods for glyphosate detection are liquid chromatography and gas chromatography coupled to mass spectrometry, which are complex, time-consuming and expensive [37,38]. This proposed system is anticipated to be a simple, cost-effective, and sensitive detection method for glyphosate.

## 2. Materials and Methods

### 2.1. Reagents

Glycerol, immobilization buffer (10 mM sodium acetate, pH 5.0), and an amine coupling kit containing 1-ethyl-3-(3-dimethylaminopropyl) carbodiimide hydrochloride (EDAC), N-hydroxysulfosuccinimide (sulfo-NHS), and 1.0 M ethanolamine · HCl, pH 8.5 (ETH) were purchased from Bio-Rad (Sundbyberg, Sweden). Glyphosate and 11-mercaptoundecanoic acid (MUA) were purchased from Merck KGaA (Darmstadt, Germany). Glyphosate receptor was obtained from Nordic-MUbio (Susteren, the Netherlands).

### 2.2. Optical Setup

**Figure 1:**
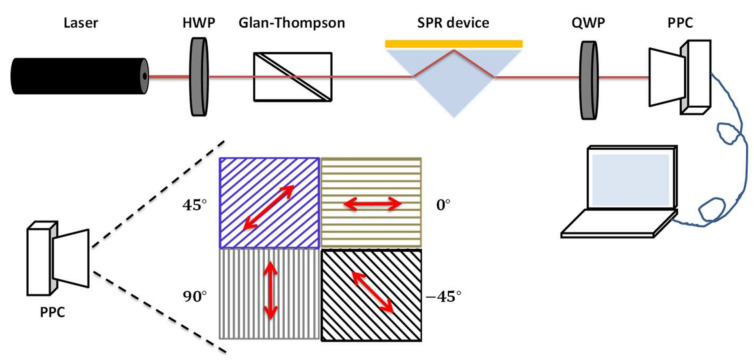
Schematic illustration of the phase-sensitive SPR based on simultaneous polarization measurement with common-path interferometry. HWP: half-wave plate, QWP: quarter-wave plate, PPC: pixelated polarization camera.

In order to measure the phase difference between the p-polarized and s-polarized components in the light beam after being affected by SPR, the phase-sensitive SPR based on simultaneous polarization measurement with common-path interferometry was established (Figure 1). A linearly polarized laser (Thorlabs Inc., Newton, NJ, USA) with a wavelength of 633 nm was used as the light source, with the output beam passing through a half-wave plate (HWP) and a Glan–Thompson polarizer to adjust the polarization for final contrast. After being incident into a homemade Kretschmann-based SPR device, the reflective light travels through a quarter-wave plate (QWP) to convert into a pair of orthogonal circular polarizations. Finally, the interference signals of the orthogonal circular polarizations were recorded by the PPC (LUCID Vision Labs, Richmond, BC, Canada). The PPC has a resolution of 2448 × 2048 and each pixel is aligned with a polarizer and a micro lens. The polarization directions of each adjacent 2 × 2 pixel are filtered at 0°, 45°, 90°, and 135°, respectively (Figure 1). Hence, light passing through the PPC can provide the intensity signal of each polarized pixel with its associated polarization information. Ultimately, the phase difference between the p-polarized and s-polarized components can be extracted from the four-polarization simultaneous measurement. The principle and algorithm of the phase extraction method used in this study are discussed in Section 3.1.

### 2.3. Preparation of the SPR Sensing Chip

The SPR sensing chips used in this study were BK7 substrates covered with Cr/Au made by the Thin Film Technology Center at the National Central University (Taoyuan, Taiwan). To detect biomolecules, the gold surface was modified with 11-mercaptoundecanoic acid (11-MUA) self-assembled monolayers (SAMs). After that, the glyphosate receptors were conjugated to the SAMs of the gold surface using amine-coupling reactions. The procedure included first activating the carboxyl group of the SAMs with the mixture of NHS (0.1 M) and EDAC (0.1 M) for approximately 10 min. Then, the glyphosate receptor at a concentration of 40 μg/mL was incubated with the reactive surface for approximately 30 min. Finally, the free active sites were blocked with ETH for approximately 7 min. Subsequently, the glyphosate in phosphate-buffered saline (PBS) solution was injected into the sensing cell to interact with the immobilized glyphosate receptors on the SPR sensing chip.

## 3. Results and Discussion

### 3.1. Principle of the Phase Extraction Method

In the proposed phase-sensitive SPR system, a linearly polarized light was introduced and incident into the SPR device. The Jones vector of the light reflected by the SPR chip surface can be expressed as

(1) $$\left[\begin{array}{c} A_{p}e^{i\phi_{p}}\\ A_{s}e^{i\phi_{s}} \end{array}\right]$$

where $A_{p}$ and $A_{s}$ are the electric field amplitudes of the reflected p- and s-polarized light, respectively; $\phi_{p}$ and $\phi_{s}$ are the corresponding phase. Since only the p-polarized component is affected by SPR, the phase of s-polarized component could be treated as a reference. Therefore, the phase change induced by SPR can be denoted as $\phi_{p}-\phi_{s}$. After the reflected light passes through the QWP, whose fast axis is at arbitrary angle θ to the *x*-axis, the Jones vector is represented by

(2) $$\left[\begin{array}{cc} \frac{1}{\sqrt{2}}+\frac{i}{\sqrt{2}}\cos\left(2\theta \right) & \frac{i}{\sqrt{2}}\sin\left(2\theta \right)\\ \frac{i}{\sqrt{2}}\sin\left(2\theta \right) & \frac{1}{\sqrt{2}}-\frac{i}{\sqrt{2}}\cos2\theta \end{array}\right]\left[\begin{array}{c} A_{p}e^{i\phi_{p}}\\ A_{s}e^{i\phi_{s}} \end{array}\right]$$

If the fast axis of the QWP forms an angle of 45 degrees to the *x*-axis (θ = 45°), the Jones vector after the QWP becomes Equation (3), which can be presented as two circular polarizations in opposite directions.

(3) $$\left[\begin{array}{c} \frac{A_{p}}{\sqrt{2}}e^{i\phi_{p}}+i\frac{A_{s}}{\sqrt{2}}e^{i\phi_{s}}\\ i\frac{A_{p}}{\sqrt{2}}e^{i\phi_{p}}+\frac{A_{s}}{\sqrt{2}}e^{i\phi_{s}} \end{array}\right]=\frac{A_{p}}{\sqrt{2}}e^{i\phi_{p}}\left[\begin{array}{c} 1\\ i \end{array}\right]+\frac{A_{s}}{\sqrt{2}}e^{i\phi_{s}}\left[\begin{array}{c} i\\ 1 \end{array}\right]$$

Next, the two circular polarizations interfere due to the use of polarization filters in the PPC. Afterward, the interference signals of the four polarization angles are acquired by the PPC at the same time. For instance, the Jones vector of the light after the polarization filter at 0° on the PPC is

(4) $$\vec{E}\left(0{}^{\circ}\right)=\left[\begin{array}{cc} 1 & 0\\ 0 & 0 \end{array}\right]\left[\begin{array}{c} \frac{A_{p}}{\sqrt{2}}e^{i\phi_{p}}+i\frac{A_{s}}{\sqrt{2}}e^{i\phi_{s}}\\ i\frac{A_{p}}{\sqrt{2}}e^{i\phi_{p}}+\frac{A_{s}}{\sqrt{2}}e^{i\phi_{s}} \end{array}\right]=\left[\begin{array}{c} \frac{A_{p}}{\sqrt{2}}e^{i\phi_{p}}+\frac{A_{s}}{\sqrt{2}}e^{i\left(\phi_{s}+\frac{\pi }{2}\right)}\\ 0 \end{array}\right]$$

In addition, the corresponding light intensity is then given as

(5) $$I\left(0{}^{\circ}\right)={\vec{E}}^{*}\left(0{}^{\circ}\right)\cdot \vec{E}\left(0{}^{\circ}\right)=\frac{A_{p}{}^{2}}{2}+\frac{A_{s}{}^{2}}{2}+A_{p}A_{s}\cos\left(\phi_{p}-\phi_{s}-\frac{\pi }{2}\right)$$

Similarly, the light intensities at the other three polarization filters on the PPC are

(6) $$I\left(90{}^{\circ}\right)=\frac{A_{p}{}^{2}}{2}+\frac{A_{s}{}^{2}}{2}+A_{p}A_{s}\cos\left(\phi_{p}-\phi_{s}+\frac{\pi }{2}\right)$$

(7) $$I\left(45{}^{\circ}\right)=\frac{1}{2}\left(A_{p}{}^{2}+A_{s}{}^{2}+A_{p}A_{s}\cos\left(\phi_{p}-\phi_{s}-\frac{\pi }{2}\right)+A_{p}A_{s}\cos\left(\phi_{p}-\phi_{s}+\frac{\pi }{2}\right)+2A_{p}A_{s}\cos\left(\phi_{p}-\phi_{s}\right)\right)$$

(8) $$I\left(-45{}^{\circ}\right)=\frac{1}{2}\left(A_{p}{}^{2}+A_{s}{}^{2}+A_{p}A_{s}\cos\left(\phi_{p}-\phi_{s}-\frac{\pi }{2}\right)+A_{p}A_{s}\cos\left(\phi_{p}-\phi_{s}+\frac{\pi }{2}\right)-2A_{p}A_{s}\cos\left(\phi_{p}-\phi_{s}\right)\right)$$

The expressions of the four light intensities ($I\left(0{}^{\circ}\right),\;I\left(90{}^{\circ}\right),\;I\left(45{}^{\circ}\right),I\left(-45{}^{\circ}\right)$) are composed of DC and interference items, and only the interference item is associated with the phase difference $\left(\phi_{p}-\phi_{s}\right)$ between the p- and s-polarized light. Finally, the phase difference is calculated by

(9) $$\phi_{s}-\phi_{p}=tan^{-1}\left(\frac{I\left(90{}^{\circ}\right)-I\left(0{}^{\circ}\right)}{I\left(45{}^{\circ}\right)-I\left(-45{}^{\circ}\right)}\right)$$

The proposed phase-sensitive SPR sensor is able to obtain the intensities of the four polarizations simultaneously by integrating common-path interferometry with the PPC, breaking down the drawbacks of the complex optical setups and asynchronous measurements.

### 3.2. Validation of the Phase Difference Calculation Method

**Figure 2:**
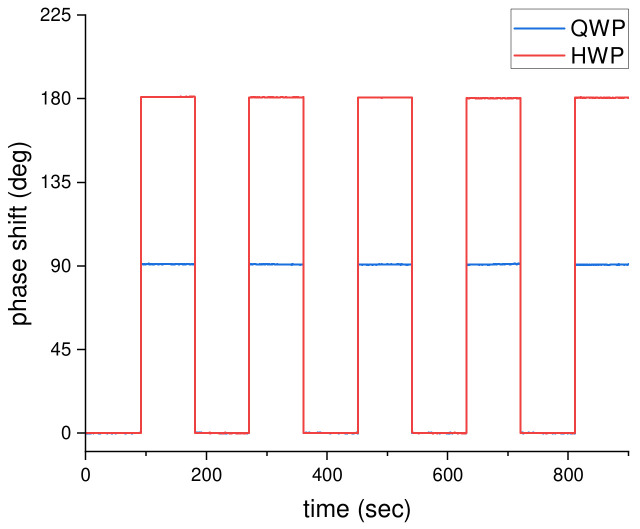
The phase shift of the QWP and HWP measured by the proposed simple phase-sensitive SPR sensor.

In this study, the phase difference between the p- and s-polarized light was determined from the polarization-dependent signals recorded by the PPC on the basis of phase-shift algorithms as discussed above. To confirm the correctness of the estimated phase difference using the PPC and the algorithms, waveplates (such as QWP and HWP) with known phase differences were measured firstly. Experimentally, by inserting the standard waveplate into the optical path (between the SPR device and the QWP, see Figure 1), a known phase retardation was introduced. Then, the resulting phase differences in p- and s-polarized light were obtained through the proposed phase extraction method. Figure 2 showed the estimated phase differences after inserting the QWP and HWP into the optical path, in which each waveplate was measured five times and the average estimated phase differences for QWP and HWP were 90.8 and 180.6 degrees, respectively. Both the estimated phase differences are very close to the theoretical phase differences, π/2 and π, of the QWP and HWP, respectively. In addition, the coefficient of variation (CV) of the estimated phase differences were less than 0.2% for the two waveplates, in which the CV value could usually be used to represent the precision or repeatability of the system. Moreover, the residual coefficient of variation (RCV) were around 1% and 0.5% for the QWP and HWP, respectively; RCV was defined as the residual standard deviation divided by theoretical phase differences of the waveplates (π/2 for QWP and π for HWP), which was used to describe the accuracy of the system in this study. The results verified that the proposed phase-sensitive SPR sensor has acceptable precision and accuracy values. 

### 3.3. Stability and Detection Performance of the Proposed Phase-Sensitive SPR Sensor

**Figure 3:**
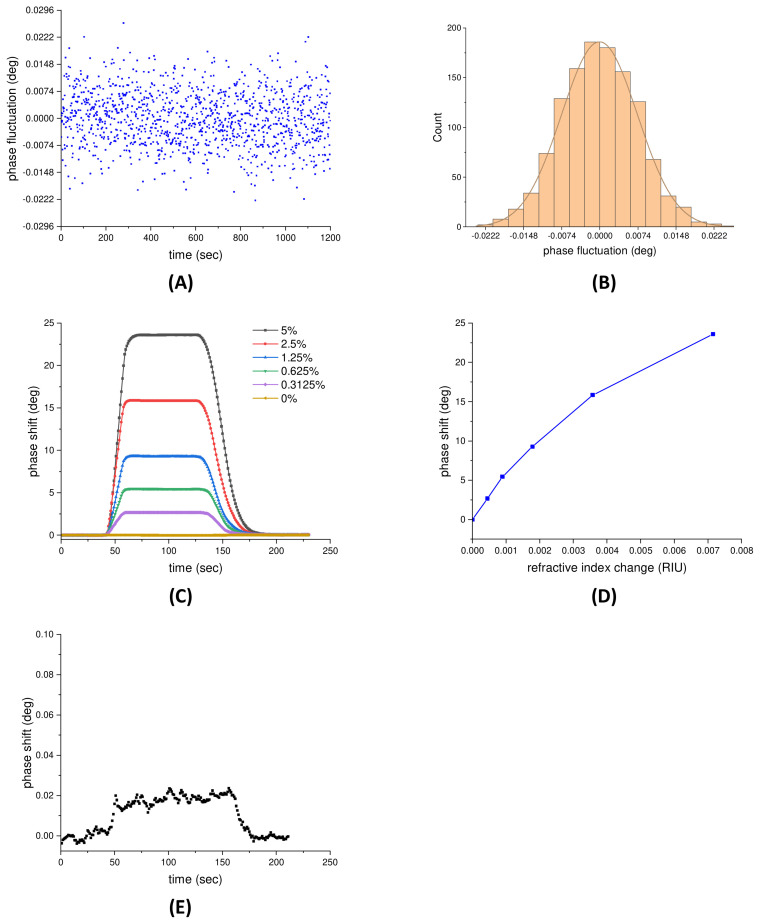
(**A**) The phase fluctuations of the system in DI water environment over a period of 20 min. (**B**) The distribution of the data points in phase fluctuation measurement. An angle of 0.0074 degrees indicates one standard deviation of the phase fluctuation. (**C**) The SPR phase shift measured by the phase-sensitive SPR sensor with respect to time for glycerol–water solution concentrations from 0% to 5%. (**D**) The relationship between the SPR phase shift and the refractive index change. (**E**) The phase shift of 0.3125%$\times \frac{1}{500}$ glycerol (8.94 × 10^−7^ RIU) measured by the proposed phase-sensitive SPR sensor.

The performance of the SPR sensors strongly depends on the noise properties of the detection system [39]. Thus, prior to the performance evaluation of the proposed phase-sensitive SPR sensor, the stability of the system should be assessed. In the proposed system, the phase shifts of SPR were obtained by the phase extraction method as described above. Experimentally, the phase stability was executed in deionized (DI) water near the SPR angle for 20 min. The phase fluctuations over a period of 60 min are shown in Figure 3A, and the standard deviation (SD) is calculated to be 7.4 × 10^−3^ degrees. In addition, Figure 3B represents the distribution of the data points; approximately 68% (823 points), 95% (1142 points), and 99.7% (1196 points) lie within 1 SD, 2 SD, and 3 SD, respectively. Therefore, the probability distribution exhibits a normal distribution, which indicates the phase fluctuations are statistically independent and dominantly white Gaussian noise. In the following, we adopt a moving average strategy to filter the noise and then increase the signal to noise ratio because, generally, if a signal contains normally distributed noise, the adjacent averaging is a good choice for removing the background noise.

The physical sensitivity to refractive index changes in the proposed phase-sensitive SPR sensor were evaluated by measuring a glycerol–water solution with concentrations ranging from 0% to 5%, corresponding to a refractive index range from 1.33095 to 1.33810 refractive index unit (RIU). These solutions were subsequently injected into the sensing cell at a constant flow rate. The SPR phase shift measured by the phase-sensitive SPR sensor with respect to time is shown in Figure 3C. The results reveal that the SPR phase shift exhibits an increasing response with raising glycerol concentration. Meanwhile, the relationship between the SPR phase shift and the refractive index change is illustrated in Figure 3D, in which error bars represent 1 SD in each measurement and are smaller than the symbol size. It can be observed a linear region with the highest slope occurs from 0 to 8.94 × 10^−4^ RIU, which represented the most sensitive region of the sensor, implying a near-resonance condition. The highest slope, i.e., the maximum sensitivity, was ~6.07 × 10^3^ degrees/RIU. Therefore, the physical detection limit, defined as the SD of the blank (DI water) divided by the maximum sensitivity of the system, was estimated to be 4.6 × 10^−7^ RIU. Taking this one step further, the 0.3125% glycerol was diluted 500-fold to obtain 8.94 × 10^−7^ RIU, close to the estimated physical detection limit, and then measured by the proposed phase-sensitive SPR sensor. The result is shown in Figure 3E, and the signal is estimated to be equivalent to seven times the baseline noise (SNR ≈ 7). An SNR ≥ 3 is considered a reliable threshold for distinguishing signal from background noise and, therefore, the physical detection limit is very close to the estimated value (4.6 × 10^−7^ RIU).

The physical detection limit of refractive index change achieved by other phase-sensitive SPR sensors is summarized in Table 1. The physical detection limit is in the range 8.0 × 10^−6^ RIU to 2.0 × 10^−8^ RIU, and most of the values are at the 10^−7^ RIU level. The proposed phase-sensitive SPR sensor was constructed with a simpler optical setup but exhibits a comparable performance. 

### 3.4. Application to Glyphosate Detection

Glyphosate is a small molecular (169.1 Da) organophosphorus herbicide and is the most widely used nonselective herbicide in the world [37,40]. In addition, glyphosate has been heavily restricted in many areas because it has perennial toxicity, residual toxicity, and chronic effects [38]. For instance, the US EPA (Environmental Protection Agency) stipulates that the maximum level of glyphosate in drinking water should be lower than 0.7 μg/mL (4.14 μM) [41]. Meanwhile, in the European Union and China, the maximum residual level of glyphosate in many crops and fruits should be lower than 0.1 μg/g (0.1 ppm) and 0.5 μg/g (0.5 ppm), respectively [42,43]. To date, the gold standard analysis approaches for glyphosate are complicated, time-consuming, and need well-trained technicians. This drives investigators to develop other ways to detect glyphosate that can bypass these issues. 

**Figure 4:**
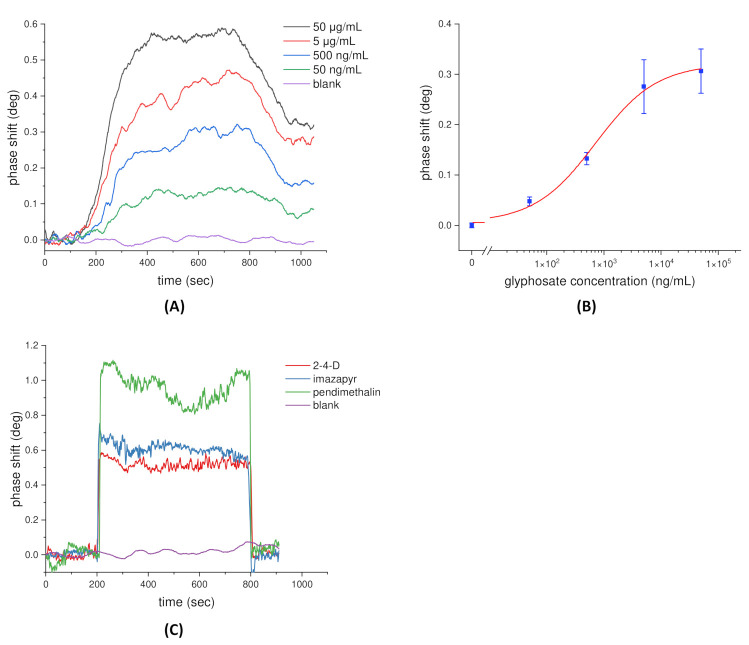
(**A**) The sensorgrams of glyphosate binding reaction measured by the phase-sensitive SPR sensor. (**B**) The standard curve of the glyphosate detection over the range of 50 ng/mL to 50 ug/mL. (**C**) Specificity test measured by the proposed phase-sensitive SPR sensor.

To detect glyphosate, the chip surface was modified with glyphosate receptors using the method described in Section 2.3. The sensorgrams of the glyphosate binding reaction ranging from 50 ng/mL to 50 μg/mL are shown in Figure 4A, which shows the SPR phase shift increasing in accordance with the increasing concentration of glyphosate. At the same time, PBS solution was the dilution buffer and also acted as the control group (blank). The sample was injected into the sensing cell and interacted with the immobilized glyphosate receptors around 100 s, and then the washing step began at approximately 700 s. The SPR phase shift with respect to the concentration of the glyphosate over the range of 50 ng/mL to 50 μg/mL is shown in Figure 4B. The standard curve of the glyphosate detection was analyzed by fitting with a nonlinear dose–response model, and the error bar indicates 1 SD in three independent measurements. In accordance with the definition of the International Union of Pure and Applied Chemistry (IUPAC), the detection limit of the proposed phase-sensitive SPR sensor for glyphosate detection was estimated to be 15 ng/mL from the experimental results. This value is almost one order of magnitude lower than the worldwide strictest residual level (0.1 μg/g or 0.1 ppm) of glyphosate. Moreover, we chose three herbicides (2,4-D, imazapyr, and pendimethalin) to further confirm the specificity of the receptor. Experimentally, the concentrations were 5, 4.6, and 5.6 μg/mL for 2,4-D, imazapyr, and pendimethalin, respectively. The results, shown in Figure 4C, revealed that after the washing step, no increment in SPR phase shifts was found in the three herbicides where the acquired phase shifts were similar to those of the blank test. This result implied that the receptor was specific to glyphosate herbicides.

## 4. Conclusions

In this study, a phase-sensitive SPR based on a simultaneous polarization measurement with a common-path interferometer configuration was developed. The SPR-induced phase difference between the p-polarized and s-polarized components was extracted from the polarization-dependent signals which were recorded by the PPC. The phase information can be easily acquired on the basis of phase-shift algorithms in a single snapshot, eliminating the drawbacks of complex optical setups and asynchronous measurements. The proposed phase-sensitive SPR sensor offers a simple and effective method of measuring the phase shift for real-time detection with no need of phase modulators. These help to reduce the complexity and the noise of the system. Moreover, this technique enables internal referencing to be implemented simpler than the traditional phase-sensitive SPR sensors; the internal referencing is helpful in degrading the effect of microfluctuations of temperature, which plays a key role in the SPR detection performance. Experimentally, a physical detection limit of 4.6×10^−7^ RIU for refractive index changes was achieved for the developed phase-sensitive SPR. In addition, this SPR sensor was applied for the detection of glyphosate, with the concentration ranging from 50 ng/mL to 50 μg/mL. The detection limit was estimated to be 15 ng/mL, which is almost one order of magnitude lower than the worldwide strictest residual level of glyphosate. Given the simple optical setup and acceptable precision and accuracy, we believe that our phase-sensitive SPR system represents a prospective method of acquiring phase signals.

## Figures and Tables

**Table 1:** The physical detection limit achieved by other approaches and the proposed SPR approach.

	Physical Detection Limit	Reference
electronic modulators	1.2 × 10^−6^ RIU~5.5 × 10^−8^ RIU	[21,22,23,24,26,27,28,29]
spectral phase modulation	8.0 × 10^−6^ RIU~2.0 × 10^−8^ RIU	[20,31,32]
rotating analyzer	10^−6^ RIU~10^−7^ RIU	[35,36]
this method	4.6 × 10^−7^ RIU	

## Data Availability

Data are contained within the article.
